# Evaluation of a new combined Western and line blot assay (EUROLINE-WB) for diagnosis and species identification of Echinococcus infection in humans

**DOI:** 10.3205/id000041

**Published:** 2019-02-19

**Authors:** Susanne Deininger, Nele Wellinghausen

**Affiliations:** 1 MVZ Labor Ravensburg, Germany

**Keywords:** echinococcosis, echinococcus, immunodiagnosis, serology, blot

## Abstract

Serological detection of echinococcosis is crucial for diagnosis and management. We evaluated the new blot assay Euroline-WB (ELB, Euroimmun) which consists of a Western blot with *Echinococcus multilocularis (E.m.)* vesicle antigens and a line blot part with recombinant antigens from *E. granulosus* (*E.g.*, genus-specific EgAgB) and *E.m.* (species-specific Em18 and Em95), in comparison to a commercial Western Blot (EWB, LDBio) for detection and species differentiation of echinococcosis within routine laboratory diagnostics. Thirty-five serum samples from 35 patients classified according to a standardized classification were included in the analysis. Out of 24 cases of proven and probable infection with *E.m.* or *E.g.* 16 (66.7%) and 15 (62.5%) were correctly identified on species level by EWB and ELB, respectively. False *Echinococcus* species were assigned in two cases by EWB but none by ELB. Negative blot results in patients with proven infections were noticed in 8.3% (ELB) compared to 4.2% (EWB), but were limited to patients with antiparasitic therapy or post-surgery indicating a treatment-induced loss of antibody activity. Thus, identification of *Echinococcus* infection at least on the genus level was possible in 23/24 (95.8%) and 19/24 (79.2%) of patients by EWB and ELB (or 22/24 patients (91.7%) including borderline results of ELB), respectively. Recombinant Em18 and Em95 were highly specific for detection of *E.m.* infection but differed in sensitivity (Em18 56% and 80 %, and Em95 22% and 20% in proven and probable infections, respectively). Advantages of ELB are the standardized analysis of the banding pattern by EUROLineScan software and a faster turn-around-time.

## Introduction

Echinococcosis is a worldwide distributed parasitic disease mainly caused by larvae of the fox tapeworm *Echinococcus (E.) multilocularis* and the dog tapeworm *E. granulosus*. While *E. granulosus (E.g.)* occurs worldwide, *E. multilocularis (E.m.)* is only prevalent on the northern hemisphere. In Europe, *E.g.* is mainly distributed in Mediterranean countries and the Middle East. In contrast, *E.m.* is endemic in central Europe, especially Germany, Switzerland, and France, but has expanded to countries further east and north in recent decades [1]. 

Alveolar echinococcosis (AE), caused by *E.m.*, is characterized by an infiltrative growing tumor, preferentially in the liver, that often forms metastasis. In contrast, larvae of *E.g.*, the cause of cystic echinococcosis (CE), grow as large cysts with brood capsules without the development of metastasis. The liver is most often affected but localization of cysts in the lung or other organs occurs more frequent than in AE. Therapeutic measures of echinococcosis differ in dependence of the kind (AE or CE) and stadium of disease, and include surgical removal of cysts, application of antiparasitic drugs, like albendazole, or even a “wait-and-watch-approach” [2], [3], [4]. An early diagnosis and subsequent treatment of affected persons may reduce complications of the disease and, thus, improve prognosis of the patients. 

The diagnosis of echinococcosis is based on imaging techniques, histopathology, and serological tests. Since imaging results can be difficult to interpret and histopathology is not achievable in all patients, serology is a helpful non-invasive tool for diagnosis of disease and monitoring of follow-up. For serological screening enzyme-linked immunosorbent assays (ELISA) and indirect hemagglutination (IHA) tests based on crude echinococcal extract from hydatid fluid of *E.g.* are widely used. For diagnosis of AE, apart from *E.-m.*-specific in-house tests an ELISA detecting *E.-m.*-specific IgG antibodies by use of recombinant *E.-m.*-specific Em2-Em18 antigens is commercially available [5]. However, serological cross reactions between other parasites and also between *E.g.* and *E.m.* do occur frequently and hamper identification of the causative *Echinococcus* species [5]. On the other hand, encapsulation of *Echinococcus* within the cysts restrains the release of antigens, thus, leading to negative test results. Antigen release is highest in liver cysts, about 50% in cases of exclusive lung cysts and even lower in central nervous system manifestations. 

For serological confirmation of echinococcosis and for differentiation between infections with *E.m.* and *E.g.* a commercially available Western blot assay (EWB, LDBio) that uses a whole larval antigen extract of *E.m*. can be used [5], [6], [7]. Recently, a new immunoblot has been brought to market (Anti-Echinococcus EUROLINE-WB (IgG), EUROIMMUN AG). This test consists of a Western blot with *Echinococcus* vesicle antigens and, in addition, a line blot part with recombinant antigens from *E.m.* and *E.g.* In the present study, for the first time we evaluated the new EUROLINE-WB (ELB) in comparison to the EWB under routine laboratory conditions. In addition, the diagnostic value of the recombinant antigens EgAgB, Em18, and Em95 included in ELB was evaluated.

## Methods

### Study population

All serum samples with ambiguous results in our standard serological workup for echinococcosis requiring confirmation by Western blot (see below) between December 2014 and August 2016 were initially included in this study (n=43 sera from 43 patients). Out of these 43 patients 35 patients were regarded as cases of echinococcosis, and were finally included in the analysis. After completion of routine serological workup including blot testing by EWB, the serum samples were stored at –20°C and tested by ELB in batches.

### Serological workup

Routine workup of *Echinococcus* serology in our laboratory consists of *Echinococcus* genus-specific ELISA (Serion classic *Echinococcus* IgG (EspE), Virion Serion, Wuerzburg, Germany), *E.-multilocularis*-specific ELISA (EmE, Bordier Affinity Products SA, Crissier, Switzerland), and IHA (Cellognost Echinococcosis, Siemens, Marburg, Germany). If these tests do not allow diagnosing or ruling out echinococcosis including species differentiation in case of positive serological results, a Western blot (EWB, LDBio Diagnostics, Lyon, France) is performed. Serum samples from patients with known echinococcosis but fulfilling the above diagnostic criteria were also included in the study.

All tests were performed according to the manufacturer’s instructions. The EspE uses inactivated hydatid fluid from *E.g.* as antigen and detects IgG against *E.g*. and *E.m*. quantitatively (<10 U/ml negative, 10–15 U/ml borderline, >15 U/ml positive). The EmE is intended for detection of IgG against *E.m.* and uses recombinant *E.-m.*-specific Em2-Em18 antigens. Results are indicated in index values (index <90 negative, 90–110 borderline, >110 positive). The IHA contains human 0-erythrocytes sensitized with *E.g.* hydatid antigens. A titer of >32 is rated as positive. Serum dilutions of 1:32 are defined as borderline and <1:32 as negative. 

The *Echinococcus* Western blot EWB contains electrophoretically separated *E. m.* larval antigen extract and detects IgG against *E.m.* and *E.g.* A positive control provided in the kit was used in each run as a reference for identification of visible bands. All visible bands are regarded as positive. According to the instructions of the manufacturer, results are classified in five different profiles: Profile P1 (7 kDa band only) and profile P2 (7 kDa band, large diffuse 17 kDa band and very often 26–28 kDa band) are specific for *E.g.* Profile P3 (26–28 kDa band and the narrow 16 and/or 18 kDa bands and often additional 7, 12, 15, 17, 20 or 24 kDa bands) is specific for *E.m.* Profile P4 (isolated 26–28 kDa band) and profile P5 (7 kDa and 26–28 kDa band) confirm echinococcosis but do not allow differentiation between *E.g.* and *E.m.*

The *Echinococcus* Euroline-WB blot (ELB) combines a Western blot and a line blot for detection of IgG against *Echinococcus*. The blot membrane consists in one part of electrophoretically separated *E.m.* vesicle fluid extract (native antigen) and in the other part of so-called membrane chips carrying the selected recombinant antigens EgAgB (specific for *Echinococcus* spp.) and Em18 and Em95 (specific for *E.m.*). In addition, a serum control band is included in each membrane and also a control band which detects the IgG conjugate used. The ELB can be evaluated by a flatbed scanner (e.g. EuroBlotscanner) followed by analyzation using the EUROLineScan software (EUROIMMUN). If the calculated intensity value is higher than the set cut-off value, a band is rated positive. Bands with calculated intensity values between 13 and 17 are rated borderline by the software. According to the manufacturer, a positive Em18 or Em95 band is specific for *E.m.*, while a positive EgAgB band can be obtained for both species. In the Western blot region 7 kDa, p16/18 kDa, p21 kDa and p25/26 kDa bands are evaluated. The blot is regarded negative if there is no band or only a positive 25/26 kDa band or a borderline EgAgB band. Borderline results are defined as a positive EgAgB band and a borderline 7 kDa, 16/18 kDa, 21 kDa, Em18, or Em95 band. The detection of at least one band out of 7 kDa, 21 kDa, 16/18 kDa, Em18, or Em95 is regarded as positive for *Echinococcus* spp. If the Em18 and/or Em95 band is detectable, *E.m.* infection is assumed. In contrast, detection of a positive EgAgB band in addition to a 7 kDa, 16/18 kDa, and/or 21 kDa band is rated as *E.g.* infection. 

### Classification of patients

Patients were classified into nine groups (Table 1 (Tab. 1)) according to epidemiological data, serological results obtained by EspE, EmE, and IHA, clinical history, and imaging results according to the classification of an expert consensus for diagnosis of cystic and alveolar echinococcosis in humans [3], [4]. This classification was used as “gold standard” for evaluation of EWB and ELB blot assays. Our classification differs in some points from the classification by Kern et al. and Brunetti et al. [3], [4] since we are a diagnostic laboratory receiving specimens from external hospitals and practitioners and, thus, had to focus on serological results. In addition, we obtained clinical data as well as imaging and histological results by the referring physicians. Many patients classified as proven or probable echinococcosis had the disease for years or decades, and were under observation and treatment at various institutions in Germany and abroad. We tried to obtain as much clinical data as possible but, nevertheless, we were not able to prove all data which would have been needed for exact classification according to [3] like imaging and histological results.

## Results

### Echinococcus genus and species identification by the two blot assays

The results of the different blot assays, EWB and ELB, in the nine defined patient groups, including qualitative results and differentiation of the *Echinococcus* species, are depicted in Table 2 (Tab. 2). 

Of the nine patients with proven *E.m.* infection, one patient was falsely identified as *E.g.* infection by EWB due to the presence of a single 7 kDa band (patient no. 4, Table 3 (Tab. 3)). The ELB showed the same banding pattern (7 kDa band only) but, according to the instructions of the manufacturer, this result is interpreted as infection as *E.spp.* without species differentiation. One patient with proven *E.m.* infection who is under antiparasitic therapy for five years was negative by ELB (no. 5) and another patient was borderline (no. 6) while EWB identified infection with *E.spp.* (Table 3 (Tab. 3)).

Of the five patients with proven *E.g.* infection, one (no. 9) was negative in both, EWB and ELB. This Turkish patient underwent nephrectomy due to echinococcal cyst in the kidney 15 years ago and had no signs of residual disease by imaging. However, EspE was still positive with 37 U/ml (Table 3 (Tab. 3)). One patient from Greece (no. 7) with presence of a single 7 kDa band in both blots showed discrepant species identification due to different identification criteria of the manufacturers (see above). The three other patients in this group also came from countries endemic for *E.g.* (Turkey, Ukraine). 

The single patient with proven *E.spp.* infection but lacking species identification (no. 32) was confirmed by EWB whereas ELB did not detect any specific bands. This patient from Austria had surgical resection of a liver lesion with histological confirmation of *E.spp.* in the past and was still under antiparasitic treatment (Table 3 (Tab. 3)). 

In a 10-year-old-girl with proven *Echinococcus* infection in the spine (no. 24) and highly positive antibody titers in IHA, EspE, and EmE definite species identification was not possible but infection with *E.g.* appeared more probable. Several bands were detected in both, EWB and ELB, but species identification of the blots differed (Table 3 (Tab. 3)). In EWB, the presence of the *E.m.*-specific 18 kDa band suggested *E.m.* infection, whereas the *E.m.*-specific bands Em18 or Em95 were not detected in ELB, thus, suggesting *E.g.* infection. This rather unusual case of echinococcosis was a German girl that had not been abroad but lives with very close contact to several dogs in her family and even sleeps with the dogs in bed. She presented with a large paravertebral cyst which rapidly regressed under albendazole therapy within a few months. Surgical resection was not done.

Of the five patients with probable* E.m.* infection, four were confirmed by EWB and ELB. In one patient (no. 22) the 7 kDa and 26-28 KDa band was detected in EWB and a borderline 7 KDa band in ELB which was interpreted as infection by *E.spp.* and equivocal *Echinococcus* infection, respectively. This 73-year-old patient had several cystic lesions in the liver without typical morphology for echinococcosis. 

Of the two cases with probable *E.g.* infection, one patient from Afghanistan showed merely a borderline result by ELB since only borderline antibodies against the 16–18 KDa antigen were detected despite high EspE and IHA titers (no. 30). In contrast, detection of a positive 7 kDa band in EWB suggested *E.g.* infection (Table 3 (Tab. 3)). The other patient with probable E.g. infection also came from a country where CE is much more common than AE (Turkey). 

Of the four patients probably infected with *E.spp.*, serum of a 70-year-old patient was negative in ELB (no.27). In contrast, EWB confirmed *E.spp.* infection due to presence of a 26–28 kDa band in this patient. EspE result was borderline and IHA weak positive and this laboratory constellation was already observed four years ago. Clinical data were unfortunately not available. Serum of patient no. 28 showed borderline 21 kDa and 25/26 kDa bands in ELB but was identified as *E.m.* infection according to 16 kDa and 26–28 KDa bands in EWB (Table 3 (Tab. 3)). This 79-year-old patient showed positive results in EspE, IHA, and EmE and had several calcified liver lesions.

In the group of cases with possible *E.spp.* infection two cases were differentiated as *E.g.* infection by EWB due to a 7 kDa band. In contrast, ELB defined these cases as a borderline (no. 33) and *E.spp.* infection (no. 26) by reason of a borderline and prominent 7 kDa band, respectively. Both patients had minor ELISA and IHA results and ambiguous cystic liver lesions. Sera of the three other patients were negative in both blots. 

Altogether, out of the 24 cases of proven or probable infection with *Echinococcus* spp. and species identification (patient groups no. 1, 2, 4, 5, and 6) 16 (66.7%) and 15 (62.5%) were correctly identified on the species level by EWB and ELB, respectively (Table 4 (Tab. 4)). While a wrong *Echinococcus* species was identified in two cases by EWB, no false identification occurred by use of ELB. However, a false negative blot result was noticed more frequently in ELB compared to EWB (8.3% versus 4.2%, see Table 4 (Tab. 4)). Thus, identification of *Echinococcus* infection at least on the genus level was possible in 23/24 (95.8%) and 19/24 (79.2%) of patients by EWB and ELB (or 22/24 patients (91.7%) including borderline results of ELB), respectively.

### Diagnostic value of recombinant antigens in ELB

In ELB three recombinant antigens, i.e. EgAgB of *E.g.* and Em18 and Em95 of *E.m.*, are included. Antibodies against EgAgB were detected in 3 of the 5 (67%) patients each with proven or probable *E.g.* infection, respectively, and also in 5 of 9 (56%) patients with proven *E.m.* infection (Table 5 (Tab. 5)). In contrast, antibodies against Em18 and Em95 were nearly exclusively detected in patients with proven or probable *E.m.* infection. While the sensitivity of Em18 was 56% in proven and 80% in probable *E.m.* infections, sensitivity of Em95 was only 22% and 20%, respectively (Table 5 (Tab. 5)).

## Discussion

Serological identification and species differentiation of *Echinococcus* infection is important for the diagnosis and management of echinococcosis patients. For serological confirmation of unclear results of screening tests as well as a primary diagnostic test the commercial Western blot EWB based on E.m. larval extract is available for years. It has a high sensitivity for detection of echinococcosis (97%), but differentiation of infections by *E.m.* and *E.g.* was possible in 76% of patients only [7]. Recently, the new immunoblot ELB has been brought to market which consists of a Western blot with *E.m.* vesicle fluid antigens and, in addition, a line blot part with recombinant antigens from *E.m.* and *E.g.* In the present study, we evaluated the diagnostic value of both blots under routine laboratory conditions.

Or results showed a higher sensitivity of EWB compared to ELB (95.8% versus 91.7% including borderline results or 79.2% when regarding borderline results as negative) for detection of *Echinococcus* infection on a genus level in patients with proven or probable AE or CE. However, the number of patients with correct identification of the causative *Echinococcus* species was comparable by ELB and EWB (62.5% versus 66.7%, see Table 4). Applying the actual interpretation criteria of EWB, which have been changed some years ago, to the banding patterns of Liance et al. [7] results in a percentage of 69% of samples with species differentiation, thus, closely corresponds to our results.

The specificity of ELB appeared higher compared to EWB since no false identifications on species level were detected by ELB in patients with proven or probable *Echinococcus* infection. In contrast, two cases out of 24 (8.3%) were falsely identified on the species level by EWB. In one of these patients (no. 4, Table 3 (Tab. 3)), the presence of a single band at 7 kDa led to false identification as *E.g.* infection, according to the instructions of the manufacturer. This interpretation criterion of the manufacturer appears discussable since the 7 kDa antigen is a genus- but not species-specific antigen of *Echinococcus* [8] and this is also stated in the instructions of the manufacturer of EWB. Remarkably, the identical banding pattern defines *Echinococcus* infection without species differentiation in ELB. This difference in interpretation of a single 7 kDa band by the two blot manufacturers led to divergent results in two other patients as well (no. 7 and 26, Table 3 (Tab. 3)). The presence of a single band at 7 kDa in patient no. 4 might be explained by the fact that the patient underwent liver surgery four years ago and is since then under albendazole therapy, thus, leading to a decrease of antigen load and a fading immune response against *Echinococcus* antigens. Tappe et al. reported that the 7 kDa, 16 kDa, and 18 kDa bands markedly decrease and vanish after surgical resection of *E.m.* lesions within four years [6]. In addition, half of the individuals with antiparasitic chemotherapy showed a decrease in all diagnostic bands within two to three years [6].

The second case with false species identification (no. 24, Table 3 (Tab. 3)) was a quite unusual case of a German child with a huge paravertebral cyst. The cyst was rapidly regressing in size under albendazole treatment, suggesting *E.g.* infection [3], and the child also lives with very close contact to several dogs in the household. Bands at 7 kDa, 17 kDa, 18 kDa, and 26–28 kDa were detected by EWB which led to the identification of *E.m.* infection. However, differentiation of bands at 16 kDa and 17 kDa was difficult in EWB. In contrast, ELB led to the diagnosis of *E.g.* infection due to positive bands at EgAgB, 7 kDa, 16–18 kDa, and 25/26 kDa. It has to be noted that in two other cases visual evaluation of EWB was hindered by a weak and doubtful 16 kDa band as well as by difficult differentiation of bands at 16 kDa and 17 kDa.

Another focus of the study was the evaluation of the three recombinant antigens EgAgB, Em18, and Em95 in ELB. According to the manufacturer, these recombinant antigens are included in the blot stripe in order to improve species differentiation between *E.m.* and *E.g.* infection. EgAgB is a polymeric protein secreted into hydatid fluid of *E.g.* and involved in the process of immune evasion. It consists of the 8 kDa, 16 kDa, and 20–24 kDa subunits and is coded by a multigenic family with at least five genetic groups, i.e. EgAgB1 to EgAgB5. It has to be taken into account that the isoforms produced by *E.g.* and others produced by *E.m.* have a homology of more than 90%, and similar antigens were also found in the genus *Taenia*. Additionally, antibodies from patients with other parasitic diseases including schistosomiasis, onchocerciasis and toxocariasis have given rise to false positive reactions when tested against EgAgB [9]. In our study, three of five cases with proven and probable *E.g.* infection each (67%) were positive for EgAgB antibodies. Interestingly, five of nine cases with proven *E.m.* infection (56%) and two of five cases with probable *E.m.* infection (40%) had a positive EgAgB signal, too (Table 5 (Tab. 5)). Therefore, the recombinant antigen EgAgB does not seem to improve specificity of ELB. Nevertheless, it might be helpful to exclude infections with other parasites which can induce positive bands at 21 kDa (and 25/26 kDa). 

The recombinant antigen Em18 has been proven as a highly specific and valuable follow-up marker of AE before. Several studies have shown that Em18 antibody levels correlate with disease progression and drop below cut-off after curative therapy [6], [10], [11]. In our study, we detected no borderline or positive signal for Em18 in CE patients or patients with unlike *Echinococcus* infection supporting the high specificity of this antigen for *E.m.* infection. In contrast, the sensitivity of Em18 was lower, reaching 56% and 80% in cases with proven and probable *E.m.* infection, respectively. The apparent difference in sensitivity between proven and probable cases of *E.m.* infection is certainly caused by the small number of patients investigated. A relation between a history of surgery of lesions or the presence and duration of albendazole therapy was not observed (data not shown). 

Recently, the antigen Em95 was discussed as promising vaccine candidate for AE similar to Eg95 in CE which induced up to 98% protection in animal intermediate hosts against challenge infection with *E.g. *eggs [12]. Immunization with recombinant Em95 induced significant protection against infection with *E.m.* eggs in mice [13]. Recombinant subunits of Em95 protein containing B- and T-cell epitopes could specifically bind antibodies in serum from AE patients [14]. Because of its immunostimulatory potency, it was supposed to be a valid candidate for optimization of specificity of the immunoblot. In our study, sensitivity of Em95 was lower than that of Em18 (22% and 20% in cases of proven and probable *E.m.* infection, respectively), but specificity was very high (100%). In all patients with positive antibodies against Em95, also antibodies against Em18 were detected (data not shown). Therefore, the inclusion of recombinant Em95 in the blot did not have a diagnostic benefit in this study with regard to sensitivity of diagnosing AE. 

The following limitations of our study should be regarded. Since we included only one serum sample per patient chosen by routine diagnostic workup in our diagnostic laboratory, the study population was heterogenic with respect to disease activity, therapeutic measures etc. and no defined control group including patients with parasitic diseases other than echinococcosis was investigated. In addition, clinical, imaging and histological data were not obtainable in detail in all patients. For instance, histological results had to be obtained from the referring physician and not from the original histology report, and histology was not done at histology departments specialized in echinococcosis. Exact histological evaluation by a specialist for echinococcosis, for instance also applying the monoclonal antibody Em2G11 [15], [16], would have been desirable and would have allowed an exacter classification of the patients. Clinical and imaging data were obtained by the referring physicians but detailed records were not available. Instead, classification of the patients had to be based primarily on serology and, thus, differs in some points from the established classification system [3], see methods section. 

In our diagnostic workup ELB showed the advantage of a faster turn-around-time than EWB (2.5 h versus 4.5 h) and a more standardized evaluation due to scan software compared to visual examination of EWB by a technician. However, variability of the blot assays cannot be evaluated since the blots were performed only once.

## Conclusion

The new Western and recombinant blot ELB proofed highly specific in the detection of *Echinococcus* infection on the species level, both in AE and CE, but slightly less sensitive than the Western blot EWB regarding detection of infection on a genus level. Advantages of ELB are, however, the standardized analysis of the banding pattern by EUROLineScan software and the faster turn-around-time. In our opinion, both blots are valuable diagnostic tools for both confirmation of ambiguous results of serological screening assays, as postulated also by Siles-Lucas [17], and species differentiation of *E.g.* and *E.m.* Their value for follow-up under antiparasitic therapy can, however, not be determined yet. In addition, it has to be considered that all serological tests are influenced by the presence of circulating *Echinococcus* antigens and, therefore, should always be evaluated in the context of disease status, progression and therapy. Histology remains the diagnostic of choice for confirmation of echinococcosis, as also reflected by the actual classification scheme [3].

## Notes

### Competing interests

The authors declare that they have no competing interests.

### Funding

Euroimmun supplied the kits of ELB for this study. 

## Figures and Tables

**Table 1:**
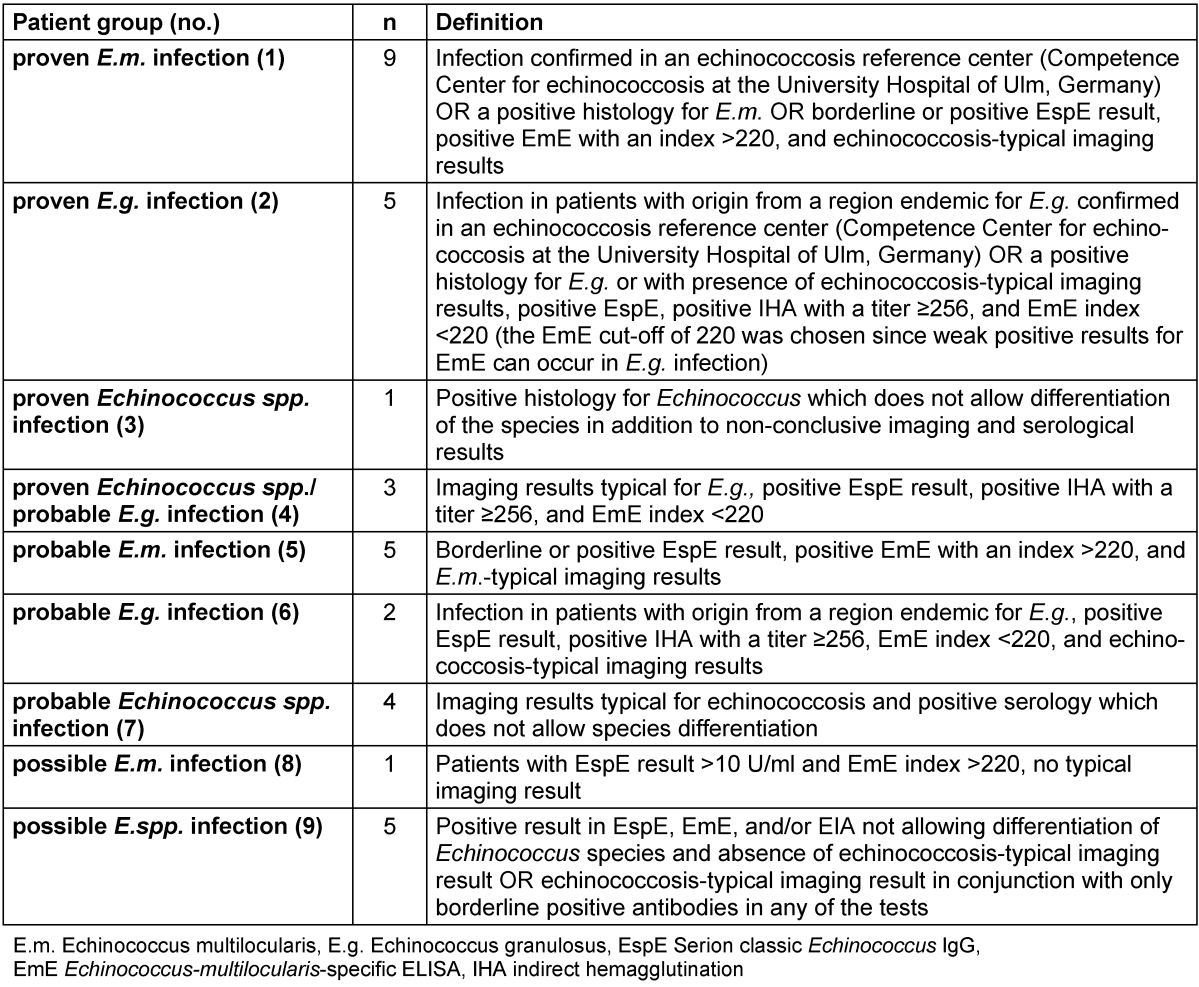
Classification of patients

**Table 2:**
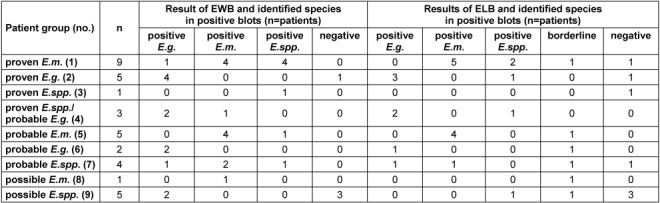
Results of blots EWB and ELB with respect to the defined patients groups

**Table 3:**
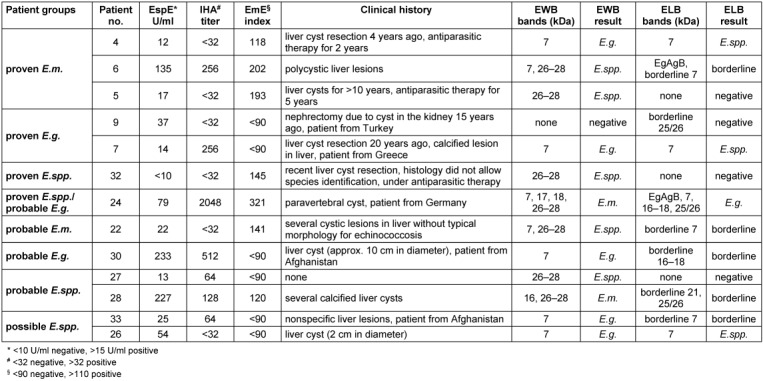
Characteristics of cases with discrepant results in EWB and ELB in comparison to the classification

**Table 4:**
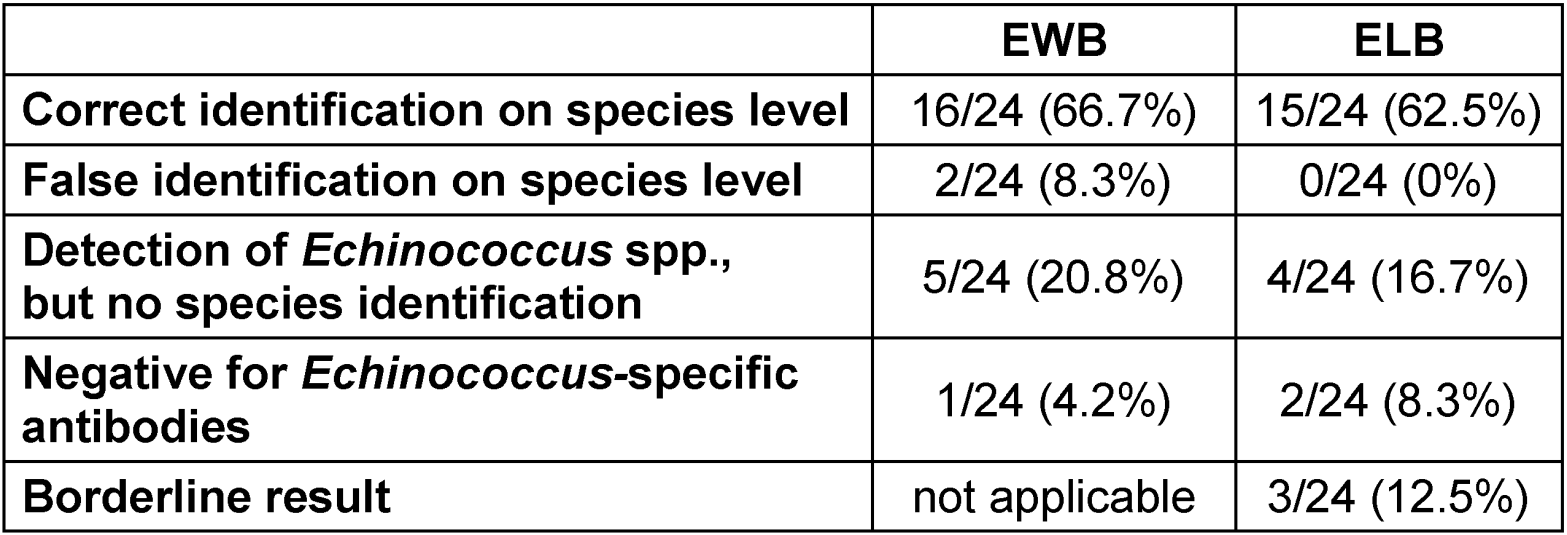
Results of EWB and ELB in all cases of proven and probable Echinococcus infection with species identification (patient groups 1, 2, 4, 5, and 6; n=24)

**Table 5:**
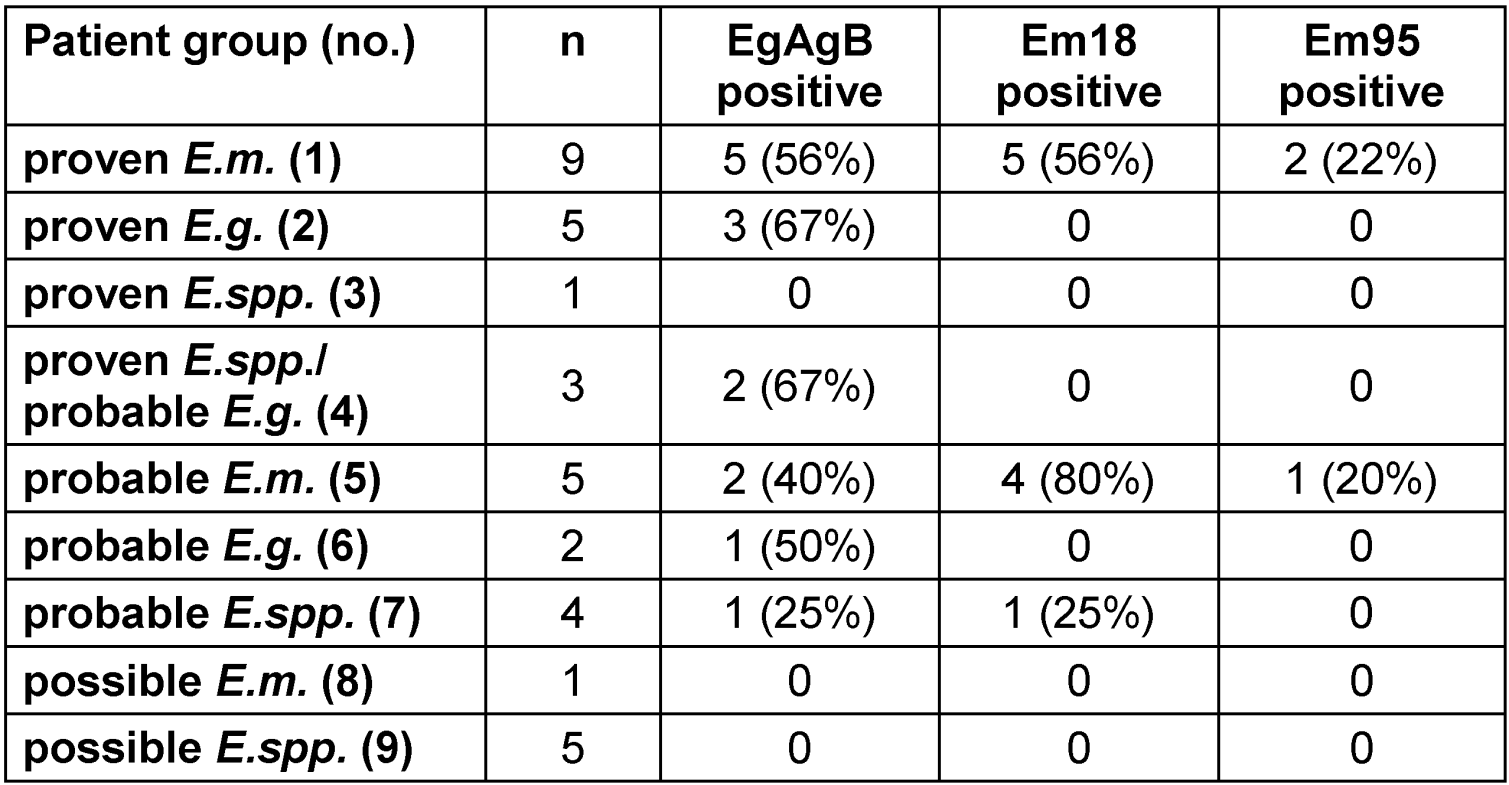
Reactivity to recombinant antigens in ELB

## References

[1] Vuitton DA, Demonmerot F, Knapp J, Richou C, Grenouillet F, Chauchet A, Vuitton L, Bresson-Hadni S, Millon L (2015). Clinical epidemiology of human AE in Europe. Vet Parasitol.

[2] Agudelo Higuita NI, Brunetti E, McCloskey C (2016). Cystic Echinococcosis. J Clin Microbiol.

[3] Kern P, Menezes da Silva A, Akhan O, Müllhaupt B, Vizcaychipi KA, Budke C, Vuitton DA (2017). The Echinococcoses: Diagnosis, Clinical Management and Burden of Disease. Adv Parasitol.

[4] Brunetti E, Kern P, Vuitton DA, Writing Panel for the WHO-IWGE (2010). Expert consensus for the diagnosis and treatment of cystic and alveolar echinococcosis in humans. Acta Trop.

[5] Reiter-Owona I, Grüner B, Frosch M, Hoerauf A, Kern P, Tappe D (2009). Serological confirmatory testing of alveolar and cystic echinococcosis in clinical practice: results of a comparative study with commercialized and in-house assays. Clin Lab.

[6] Tappe D, Grüner B, Kern P, Frosch M (2008). Evaluation of a commercial Echinococcus Western Blot assay for serological follow-up of patients with alveolar echinococcosis. Clin Vaccine Immunol.

[7] Liance M, Janin V, Bresson-Hadni S, Vuitton DA, Houin R, Piarroux R (2000). Immunodiagnosis of Echinococcus infections: confirmatory testing and species differentiation by a new commercial Western Blot. J Clin Microbiol.

[8] Ito A, Ma L, Schantz PM, Gottstein B, Liu YH, Chai JJ, Abdel-Hafez SK, Altintas N, Joshi DD, Lightowlers MW, Pawlowski ZS (1999). Differential serodiagnosis for cystic and alveolar echinococcosis using fractions of Echinococcus granulosus cyst fluid (antigen B) and E. multilocularis protoscolex (EM18). Am J Trop Med Hyg.

[9] Manzano-Román R, Sánchez-Ovejero C, Hernández-González A, Casulli A, Siles-Lucas M (2015). Serological Diagnosis and Follow-Up of Human Cystic Echinococcosis: A New Hope for the Future?. Biomed Res Int.

[10] Sako Y, Tappe D, Fukuda K, Kobayashi Y, Itoh S, Frosch M, Grüner B, Kern P, Ito A (2011). Immunochromatographic test with recombinant Em18 antigen for the follow-up study of alveolar echinococcosis. Clin Vaccine Immunol.

[11] Tappe D, Frosch M, Sako Y, Itoh S, Grüner B, Reuter S, Nakao M, Ito A, Kern P (2009). Close relationship between clinical regression and specific serology in the follow-up of patients with alveolar echinococcosis in different clinical stages. Am J Trop Med Hyg.

[12] Lightowlers MW, Lawrence SB, Gauci CG, Young J, Ralston MJ, Maas D, Heath DD (1996). Vaccination against hydatidosis using a defined recombinant antigen. Parasite Immunol.

[13] Gauci C, Merli M, Muller V, Chow C, Yagi K, Mackenstedt U, Lightowlers MW (2002). Molecular cloning of a vaccine antigen against infection with the larval stage of Echinococcus multilocularis. Infect Immun.

[14] Wang H, Zhang F, Ma X, Ma H, Zhu Y, Liu X, Zhu M, Wen H, Ding J (2014). Prokaryotic expression and identification of B- and T-cell combined epitopes of Em95 antigen of Echinococcus multilocularis. Int J Clin Exp Pathol.

[15] Barth TF, Herrmann TS, Tappe D, Stark L, Grüner B, Buttenschoen K, Hillenbrand A, Juchems M, Henne-Bruns D, Kern P, Seitz HM, Möller P, Rausch RL, Kern P, Deplazes P (2012). Sensitive and specific immunohistochemical diagnosis of human alveolar echinococcosis with the monoclonal antibody Em2G11. PLoS Negl Trop Dis.

[16] Deplazes P, Gottstein B (1991). A monoclonal antibody against Echinococcus multilocularis Em2 antigen. Parasitology.

[17] Siles-Lucas M, Casulli A, Conraths FJ, Müller N (2017). Laboratory Diagnosis of Echinococcus spp. in Human Patients and Infected Animals. Adv Parasitol.

